# A linkage disequilibrium-based approach to position unmapped SNPs in crop species

**DOI:** 10.1186/s12864-021-08116-w

**Published:** 2021-10-29

**Authors:** Seema Yadav, Elizabeth M. Ross, Karen S. Aitken, Lee T. Hickey, Owen Powell, Xianming Wei, Kai P. Voss-Fels, Ben J. Hayes

**Affiliations:** 1 Queensland Alliance for Agriculture and Food Innovation, Queensland Bioscience Precinct, 306 Carmody Rd., St. Lucia, Brisbane, Queensland 4067 Australia; 2 Agriculture and Food, CSIRO, Queensland Bioscience Precinct, St. Lucia, Brisbane, Queensland 4067 Australia; 3 Sugar Research Australia, Mackay, QLD 4741 Australia

**Keywords:** Genetic map, Linkage disequilibrium, Single nucleotide polymorphism

## Abstract

**Background:**

High-density SNP arrays are now available for a wide range of crop species. Despite the development of many tools for generating genetic maps, the genome position of many SNPs from these arrays is unknown. Here we propose a linkage disequilibrium (LD)-based algorithm to allocate unassigned SNPs to chromosome regions from sparse genetic maps. This algorithm was tested on sugarcane, wheat, and barley data sets. We calculated the algorithm’s efficiency by masking SNPs with known locations, then assigning their position to the map with the algorithm, and finally comparing the assigned and true positions.

**Results:**

In the 20-fold cross-validation, the mean proportion of masked mapped SNPs that were placed by the algorithm to a chromosome was 89.53, 94.25, and 97.23% for sugarcane, wheat, and barley, respectively. Of the markers that were placed in the genome, 98.73, 96.45 and 98.53% of the SNPs were positioned on the correct chromosome. The mean correlations between known and new estimated SNP positions were 0.97, 0.98, and 0.97 for sugarcane, wheat, and barley. The LD-based algorithm was used to assign 5920 out of 21,251 unpositioned markers to the current Q208 sugarcane genetic map, representing the highest density genetic map for this species to date.

**Conclusions:**

Our LD-based approach can be used to accurately assign unpositioned SNPs to existing genetic maps, improving genome-wide association studies and genomic prediction in crop species with fragmented and incomplete genome assemblies. This approach will facilitate genomic-assisted breeding for many orphan crops that lack genetic and genomic resources.

## Background

The rate of genetic gains in crop breeding programs can be accelerated using genomic information, either through genomic selection (GS), the use of markers linked to causal mutations of moderate to large effects discovered through genome-wide association (GWAS) if these exist, or a combination of both [1]. Ideally, the position of the markers in the genome of the target species would be known, particularly for GWAS and implementing genomic prediction exploiting epistasis [2]. Unfortunately, many current genome assemblies are fragmented or incomplete, particularly for crop species with highly complex genomes such as sugarcane [3].

Single-nucleotide polymorphisms (SNPs) are the most common polymorphisms at the DNA level. SNPs are cost-effective and easy to genotype in a high throughput manner, making them increasingly relevant in crop/animal genetics studies such as association mapping, marker-assisted, and genomic selection. With advancements in high-throughput genotyping, high-density SNP arrays are now available as an effective genetic tool for many important crop species such as barley, sugarcane, and wheat [4–7]. This has culminated in the availability of an increasing number of SNPs in these species.

Despite the development of many tools for generating genetic maps, the exact genome location of many SNPs from these SNP arrays is unknown. Sugarcane’s highly heterozygous and primarily autopolyploid genetics have all hampered the development of a comprehensive genetic map. Modern sugarcane cultivars present a high (~ 8–16) ploidy level with mainly random chromosome pairing and significant inbreeding depression, making it difficult to generate more traditional experimental mapping populations such as recombinant inbred lines or double haploids [5, 8, 9]. Moreover, the presence of single-dose and multi-dose alleles, as well as uneven chromosome numbers in the various homo (eo) logy classes due to aneuploidy, has restricted genetic mapping [8, 10]. To date, for example, all of the sugarcane genetic maps generated have had low genome coverage and limited information on the genomic organisation. One of the key reasons for this is the small number of markers that have been mapped [8].

Goddard and Meuwissen [11] proposed the idea that the chromosomal position of a quantitative trait locus (QTL) can be identified using linkage disequilibrium (LD) information from other markers with known positions across the genome. LD quantifies the non-random (statistical) association between alleles at distinct loci and represents the fundamental basis for many methods used in statistical genetics and breeding. Marker-assisted selection and genomic selection both exploit LD between markers and QTL. In modern sugarcane breeding, the use of a small number of parental clones in hybridisation schemes has reinforced significant LD, although to varying degrees depending on the populations studied [12–14].

Inferring chromosomal positions using LD can be challenging, as LD between unlinked markers can result from factors other than physical proximity on a chromosome (linkage), such as epistatic interactions, genetic drift, selection, and mutation. In addition, admixing genetically distinct populations result in the linkage between two loci with different allele frequencies, even though they are unlinked [15]. Population stratification and cryptic relationship within a population can also cause LD, resulting in correlated allele frequencies [15, 16]. Therefore, if LD estimates are used to investigate the linkage-based association, multi-point LD is less likely to be affected by the above than single point LD estimates.

Estimates of LD have been used to infer the position of the unmapped markers in diploid species [11]. Miller et al. [17] successfully demonstrated the use of an LD-based approach to map a test set of SNPs onto an existing bovine map backbone. Later, Khatkar et al. [18] used a test set of SNPs which they assigned to chromosomes and positions within chromosomes, called Locus Ordering by Dis-Equilibrium (LODE). The method was then used to allocate positions to 4688 (out of 5314) unassigned SNPs on an early bovine genome assembly (Btau4.0). Finally, the order of mapped SNPs was validated across the genome to assess genome assembly quality. The authors concluded that the LD-based approach was an accurate and efficient technique for positioning unassigned SNPs with minor allele frequency (MAF) > 0.01.

The main aim of this study was to i) adopt an LD-based algorithm to allocate unassigned SNPs in order to develop a method for expanding established genetic maps for several crops with complex genomes and to ii) investigate the accuracy of this approach in sugarcane, wheat and barley. To achieve this, a modified multi-point LODE approach was implemented. The algorithm’s efficiency was first investigated on a breeding population of sugarcane clones using a 20-fold cross-validation process. Test sets of 200 out of 4502 mapped SNPs of the newly developed sugarcane Q208 genetic map were positioned based on our modified LODE approach in every iteration. To validate the LD-based approach, the algorithm was also assessed in hexaploid wheat diversity panel, including elite varieties with a genome size estimated at 17 Gb and a structured nested association mapping population for barley, a self-pollinated and one of the largest diploid genomes (haploid genome size 5.3 Gb). Finally, the algorithm was used to assign 5920 out of 21,251 unassigned SNPs (MAF > 0.01) to the current Q208 sugarcane genetic map. This updated Q208 sugarcane genetic map is available to the sugarcane research community.

## Results

### The extent of LD and LD decay

**Fig. 1:**
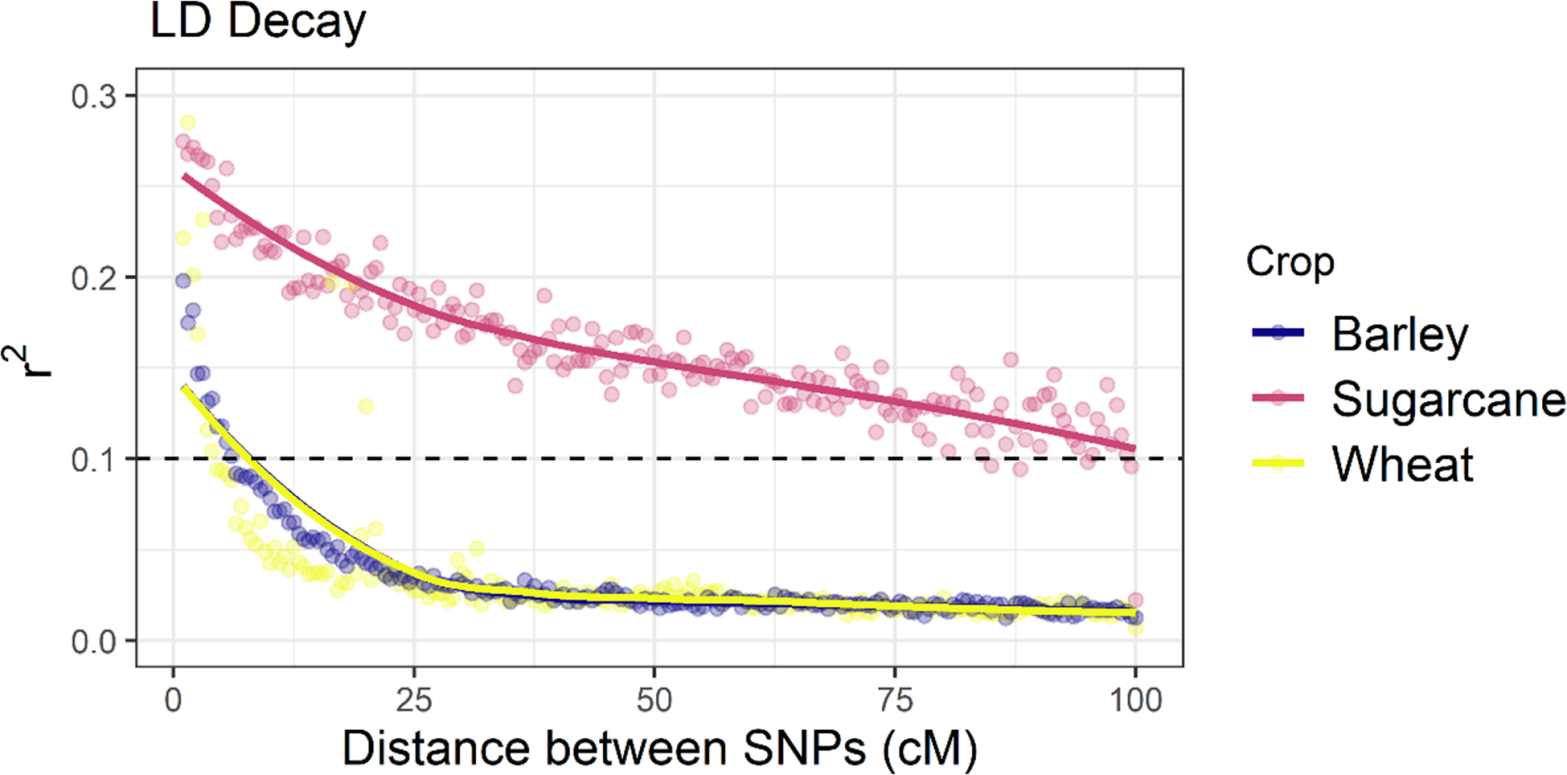
Intra-chromosomal Linkage Disequilibrium decay in elite sugarcane clones, wheat diversity panel, and structured barley lines. Estimates of LD between marker pairs in terms of correlation (*r*^2^) (after giving numerical values to allele states). LD threshold (cutoff) of 0.1 is indicated with a black dotted line. The genetic distances (cM) are from the respective genetic map, and markers having more than 100 cM are excluded from the analysis. The cutoff is considered the minimum threshold for a significant association between pairs of loci

Intra-chromosomal pair-wise LD decay between sugarcane, wheat, and barley was compared. The threshold *r*^2^ (= 0.1) is in the 75th percentile (of observed *r*^2^ value) for sugarcane, whereas it is in the 90th and 95th percentiles for wheat and barley. Sugarcane exhibited a high LD level, and LD decay was relatively slow compared to wheat and barley (Fig. 1). LD decay in wheat and barley was highly similar (9–10 cM).

### Algorithm’s accuracy

**Fig. 2:**
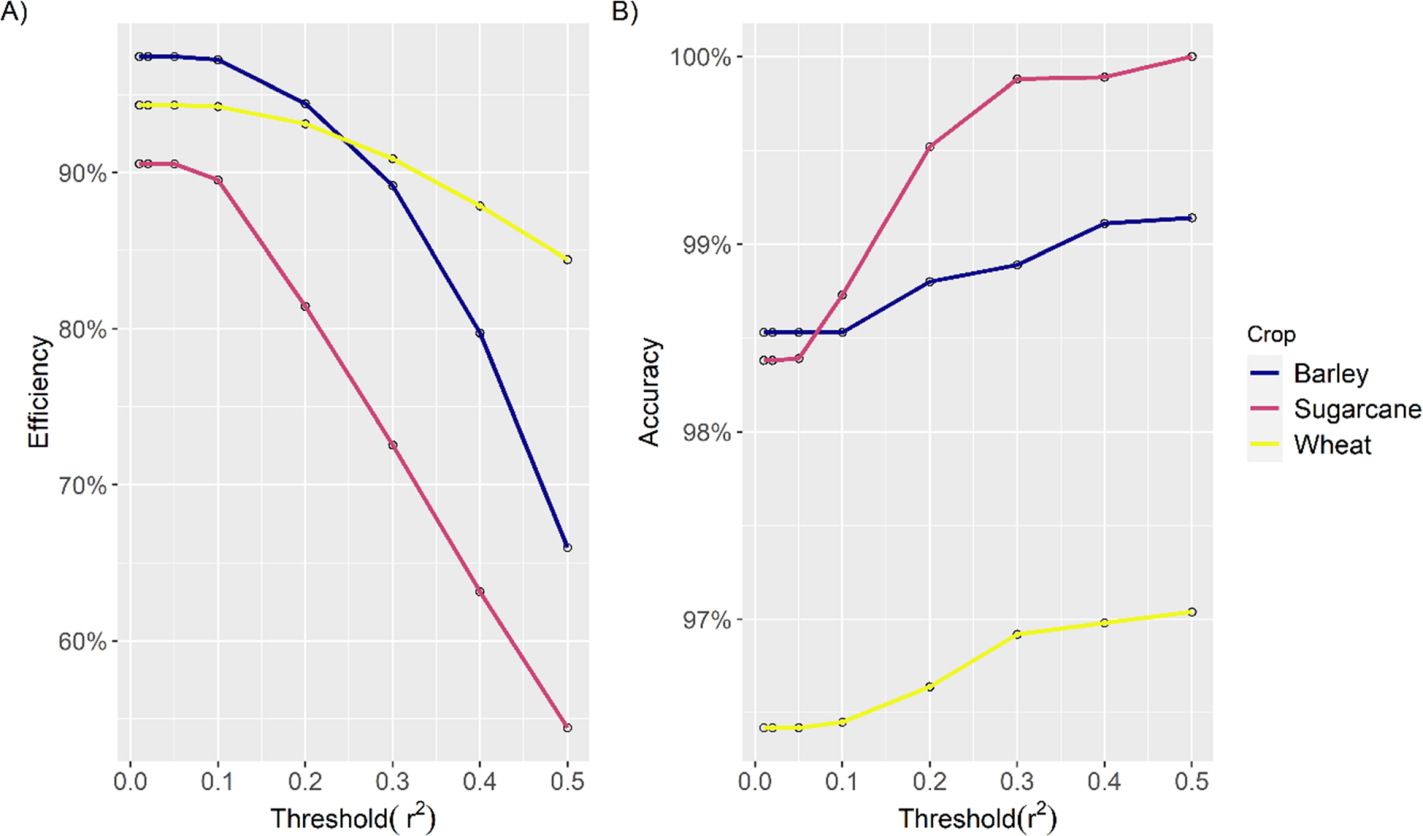
Comparison of efficiency (A) and accuracy (B) of the linkage disequilibrium- approach for placing SNPs with known position using a range (from 0.01 to 0.5) of *r*^2^ thresholds. Efficiency was defined as the proportion of SNPs placed, and accuracy was defined as the proportion of SNP markers placed on the correct chromosome

20-fold cross-validation of the LD-based algorithm was performed by defining a test subset of SNPs as unmapped. A range of *r*^2^ thresholds was tested to get the preferred threshold suitable for sugarcane, wheat and barley (Fig. 2). After setting the threshold (*r*^2^ = 0.2), the mean efficiency rate significantly decreased compared to a cutoff of *r*^2^ = 0.1 for sugarcane and barley (Fig. 2A), but the mean accuracy increased slightly (Fig. 2B). For an *r*^2^ threshold of 0.1, at least 86% of the 200 test SNPs with MAF ≥ 0.01 were assigned to a linkage group in sugarcane, while 92% of the overall SNPs could be assigned to the linkage group in some particular cross-validation scenarios, with a mean efficiency of 89.53% (Fig. 2A).

**Fig. 3:**
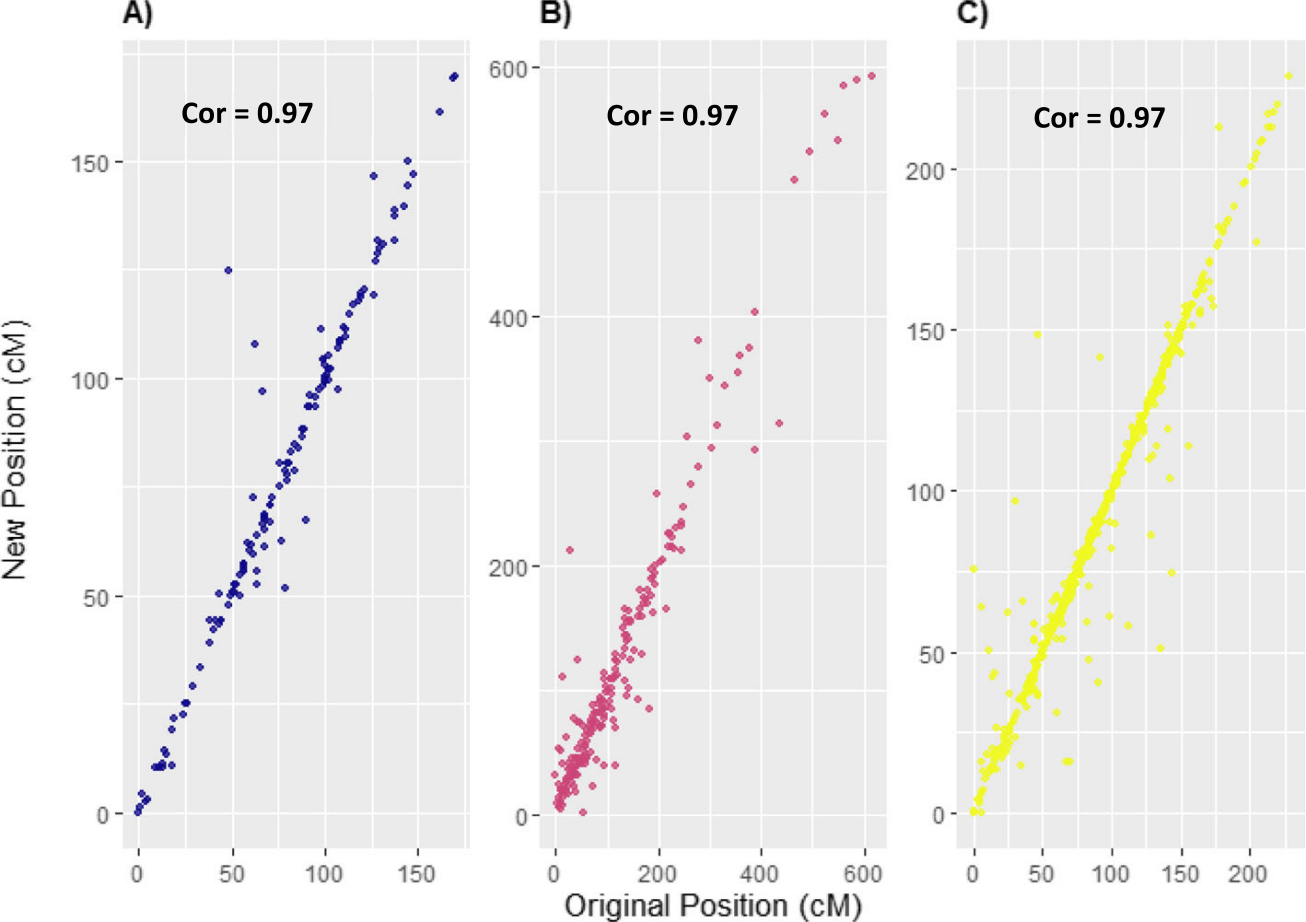
The correlation between the original and newly assigned position of one random cross-validation set for barley (A), sugarcane (B), and wheat (C). The LD based approach was used to position a known set of markers on each of the reference genomes. The assigned position was compared with the original position on the genetic map

**Fig. 4:**
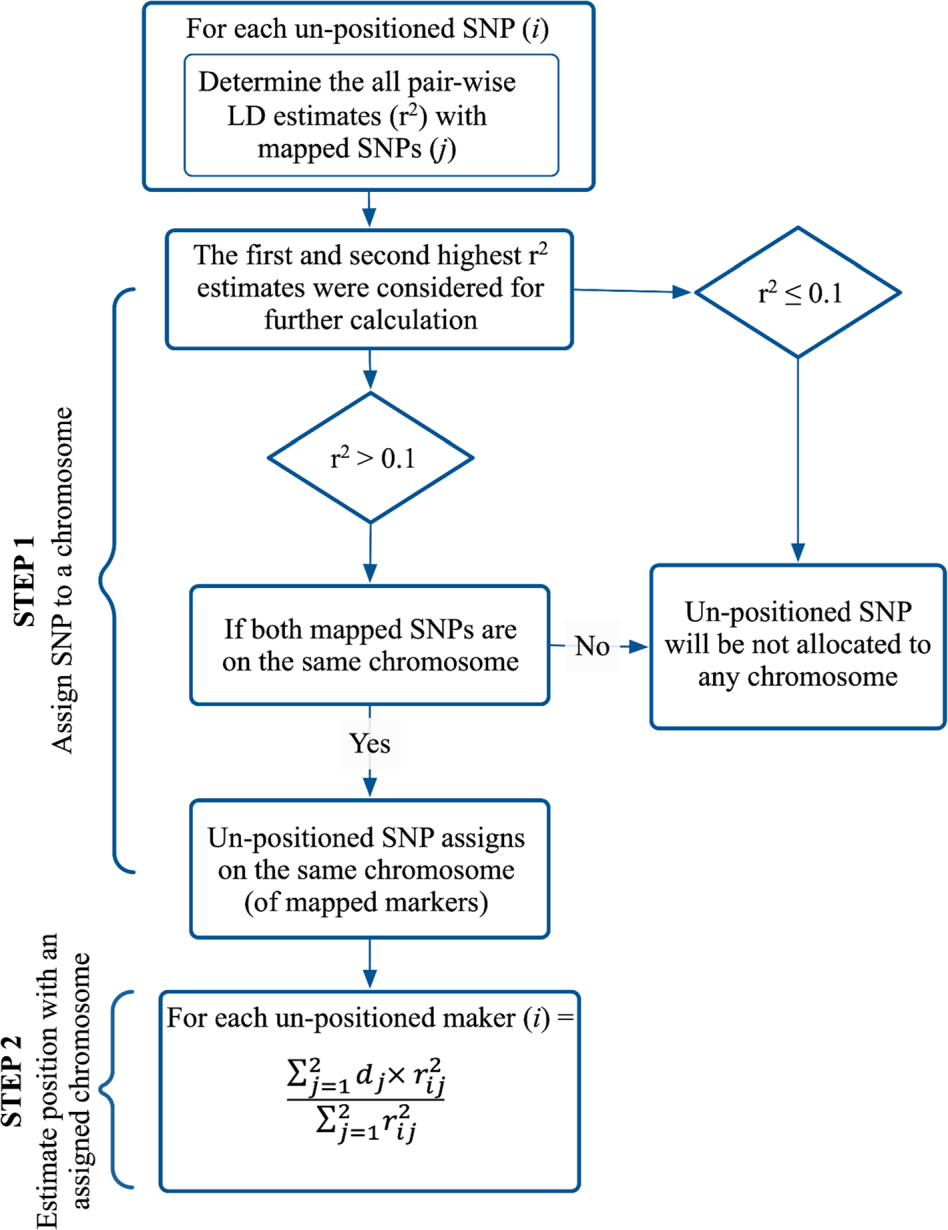
Linkage Disequilibrium based algorithm to position unassigned SNPs on the existing genetic map

For sugarcane, in most cross-validation sets, the mean accuracy in placing SNP on the right linkage group was 98.73% (Fig. 2B). The mean efficiency rate was greater in wheat (~ 94.3%) and barley (~ 97.2%) than in sugarcane (89.5%) (Fig. 2). The accuracy was comparable for all three crops. For *r*^2^ ≥ 0.4, sugarcane had a near-perfect accuracy of 100% with only a 54% efficiency. Finally, *r*^2^ ≥ 0.1 was fixed as a threshold to allocate the unassigned SNPs to the cultivar Q208 genetic map (Fig. 4). The mean distance between the original SNP position and the newly assigned position was calculated across the 20 fold cross-validation. The mean (± standard deviation) of the distance between the true and new position of the SNPs was 15.6 ± 22.6 cM, 1.1 ± 3.9 cM, and 2.7 ± 5.9 cM for sugarcane, wheat, and barley. Figure 3 depicts the relationship between the known and newly estimated positions for one random cross-validation set for the three crop species. The mean Pearson’s correlation between established and estimated SNP positions for the 20-fold cross-validation sets was found to be high, with values of 0.97, 0.98, and 0.97 for sugarcane, wheat, and barley, respectively.

### Application of LD-based approach to unpositioned SNPs in sugarcane

As a result of high throughput genotyping, approximately 25,753 high polymorphic markers with MAF (≥ 0.01) are available in a large population of approximately 3006 elite clones from the Australian sugarcane breeding program run by Sugar Research Australia. However, only 4502 markers were mapped on the newly developed Q208 genetic map (CSIRO unpublished data). Therefore, the LD-based approach (with *r*^2^ ≥ 0.1) was used to attempt to assign the remaining 21,251 unpositioned SNPs with MAF > 0.01 to the Q208 genetic map. As a result, 5920 unpositioned markers on the Q208 genetic map were successfully allocated to the existing linkage groups, resulting in a total of 10,387 SNPs with MAF > 0.01 on the extended genetic map produced with our LD-based approach.

## Discussion

This study validated an LD-based approach for efficiently and accurately mapping unassigned SNPs on genetic maps of crop species with complex genomes. An elite sugarcane population with high ploidy, a structured NAM population of diploid barley and, an allohexaploid wheat diversity panel with strictly diploid-like meiotic behaviour were used to evaluate the algorithm. Using 20-fold cross-validation, the mean proportion of mapped SNPs that were assigned to a chromosome was 89.53, 94.25, and 97.23%, whereas of the markers that were placed in the genome, 98.73, 96.45 and 98.53% of the SNPs were positioned on the correct chromosome for sugarcane, wheat, and barley, respectively.

Although LD refers to a correlation between alleles, research on LD has been confined to diploid species. There has, however, been no comprehensive investigation of LD in polyploids [19]. All current strategies for measuring LD in polyploids account for exact ploidy level (for example, 4 for tetraploid and 6 for hexaploid species), and these approaches do not indicate the level of uncertainty in their LD estimates [19–21], which is inappropriate for sugarcane owing to its varying ploidy (aneuploidy). Unlike previous sugarcane studies that used categorical association tests, such as Fisher’s exact test, to create two-way tables based on known genotypes using AFLP and RFLP markers [13, 14], we called SNP genotypes using a pseudo-diploid model due to its simplicity of implementation. This parameterisation does not consider the allele dosages, and all heterozygotes are assigned to the same genotypic class. In high polyploid species, the diploid model may result in an underestimation of heterozygosity, which is linked with advantages such as heterosis [2].

Yang et al. [12] used high-quality genome-wide SNPs identified from deep sequenced targeted regions to conduct LD analysis on three subpopulations, *S.officinarum*, *S.spontaneum*, and modern sugarcane hybrids. The correlation coefficients (*r*^2^) were estimated as a measure of LD while allelic dosage was taken into account. In population genetic studies of the highbush blueberry population, the effects of diploid and tetraploid marker genotyping calling approaches were examined. LD and population structure were found to be consistent independent of the ploidy model [22]. The same results were reported for sweet potato, a hexaploidy species [23]. This supports our choice of the diploid model and suggests that estimated LD values for sugarcane approximate real estimates. It is further backed by the fact that we accurately positioned unmapped (masked) SNPs using this method, as demonstrated by 20 fold cross-validations.

The effectiveness of the LD-based procedure for placing unassigned SNPs is determined by the magnitude of LD in the population. The extent of LD and its decay with genetic distance determines the mapping resolution with this approach and further helps determine the appropriate number of SNP markers required in association mapping studies. Moreover, both genomic and marker-assisted selection also exploits LD between markers and QTL [24, 25]. Sugarcane exhibits higher LD than many other crop species [12], which is consistent with our findings, reflecting that an elite breeding population was used in this study. In outcrossing crop species, LD is reported to decay over a short distance; however, in sugarcane, which is a perennial or vegetatively propagated crop with a long breeding cycle and a small number of historical recombination events, LD decays relatively slowly, despite the outcrossing nature of the crop [26]. The degree of LD in sugarcane may be exaggerated due to its complex ploidy, and such a high LD extent suggests that high marker density is not required for genetic studies such as GWAS and genomic selection, but it would make gene fine mapping or even map-based cloning studies difficult due to linkage drag [12, 13]. As a result, a large number of markers that can cover the entire genome are still needed to detect genomic regions within the sugarcane genome containing genes linked to desired traits [8].

Miller et al. [17] used LD estimates and a genetic algorithm approach with a minimal threshold (*r*^2^ > 0.4) to position the mapped bovine SNPs. Applying such a threshold in our study would have reduced efficiency to 63.14% in sugarcane (MAF > 0.01) and 87.9 and 79.7% in wheat and barley (MAF > 0.1), respectively. In comparison to using threshold (*r*^2^ > 0.1), Khatkar et al. [18] also recorded a decline in efficiency (71% for SNPs with MAF > 0.05) as well as a marginal drop in algorithm’s accuracy. However, our results show a slightly higher accuracy when the threshold is set (*r*^2^ > 0.4). In addition, a higher efficiency rate was observed in wheat and barley than in sugarcane, which might be because we only used SNPs with MAF > 0.1 in both species and because fewer markers had known positions in our sugarcane data set compared to the wheat and barley data sets.

Finally, we used the LD-based algorithm on sugarcane unpositioned SNPs (MAF > 0.01) by setting a threshold (*r*^2^ > 0.1) because of the high efficiency and comparable accuracy rate. However, despite the high efficiency rate, only 5920 unpositioned markers were assigned to the genetic map. This might be because only 17% of mapped single-dosage markers were used as an anchor to assign the remaining 83% of unmapped markers. The low-density anchoring to the sugarcane genome can occur due to uneven marker coverage across the genetic map, as demonstrated in all previously reported maps, whether generated via selfing or biparental crosses [5, 8, 27]. Furthermore, we used a very conservative approach considering the complex genetic inheritance, and highly heterozygous outbred parents lead to the complex segregation patterns of genotypes, which might be another reason for reduced efficiency in practical use. The algorithm described here complements other commonly used map generation methods, such as physical, radiation hybrid, and linkage mapping [27–29]. Our approach is straightforward to implement, and there have some benefits over other strategies, which are typically time-consuming, require highly specialised resources, and yield a limited resolution. It should be noted, however, that although the LD-based algorithm offers a high degree of accuracy in our data sets, it only provides an approximation of the exact position of SNPs within a chromosome because the algorithm relies on the accuracy of the genetic map that is used as a reference.

## Conclusions

The LD-based method proposed in this study might be a useful tool for placing unassigned SNPs onto current genetic maps prior to the release of the completed reference genome of crop species with complex genomes. In addition, this approach would facilitate genomic-assisted breeding for many orphan crops that lack genetic and genomic resources, which hamper the further utilisation of modern crop improvement tools such as genomic selection and genome-wide association studies.

## Methods

### Genotypic data and position of SNPs

This study employed pre-existing genotypic data from three distinct species: sugarcane [2], wheat [30], and barley [31], which were genotyped using three different platforms: Affymetrix, Illumina, and Diversity array technologies, respectively. We opted for a diverse set of species to evaluate the LD-based approach for assigning unmapped SNPs, with barley being a diploid species and wheat and sugarcane are complex allo- and auto-polyploid species.

#### Sugarcane data

For sugarcane, a 70 K SC-Affymetrix Axiom cane SNP array includes 58,028 SNP markers (primarily single or low-dosage markers) [32], was used to call genotypes for 3006 elite sugarcane clones. All heterozygous genotypes were regarded as one genotypic class in a pseudo-diploid genotyping calling model, similar to genotype calling techniques employed in prior genomic studies in sugarcane [2, 33, 34]. SNP data were classified as 0 and 2 for homozygous for the reference and alternate alleles, respectively, and 1 for the heterozygous genotype. Aitken et al. [4] provide detailed information on the cane array and genotyping calling. SNPs with a higher call rate (> 90%) were chosen for inclusion in the final dataset, yielding a total of 25,573 high-quality SNPs with MAF > 0.01 in 2909 clones. The position of 4502 (out of 25,573) SNPs has been assigned to a new Australian cultivar Q208 genetic map (CSIRO, unpublished data), and the rest of the SNPs were categorised as “unassigned” markers in the genome.

#### Wheat data

An international diversity set of 460 hexaploid wheat accession, including elite varieties, landraces, and experimental lines from different geographic backgrounds, was genotyped using a 90 K SNP wheat genotyping array (Illumina Inc.) [30]. For this study, markers with more than two alleles, MAF (< 0.1) and missing data (> 0.1), were excluded from the raw marker data. Finally, the 450 genotypes yielded 18,475 high-quality polymorphic SNPs with known map positions on the consensus map described by Wang et al. [6].

#### Barley data

The multi-parent nested association mapping (NAM) lines utilised in the validation study were derived from crossing the three Australian reference varieties Commander, Compass, and La Trobe to donor parents, which are elite breeding lines from the Northern Region Barley Breeding Program [31]. The NAM population comprises families of 50–60 lines derived from each reference variety × donor line cross. A total of 1345 F4:F6 NAM lines were genotyped with DArT-Seq markers using Barley PstI (BstNI) v1.7 array. The centimorgan (cM) positions of individual markers were projected for the Bowan DArT-Seq genetic map. Only markers that have mapped positions in the consensus map [31, 35] were used in our study, culminated in 2631 high-quality polymorphic DArT markers with MAF > 0.1.

### Estimation of linkage disequilibrium

Estimates of LD were obtained as *r*^2^ statistics (square of the correlation coefficient) for all pair-wise combinations of SNPs in each of the crop species mentioned above using a function st.calc.ld (ld.measure = “*r*^2^”) implemented in the R package “SelectionTools” version 19.3 (population-genetics.uni-giessen.de/ ~ software /). The option “ld.measure = “*r*^2^” assume that the input data is in the correct (known) gametic phase and estimates the correlation between two variables, coded as 0, 1, and 2, which indicate the number of alternative alleles at each SNP, based on genotype allele count (without phasing). For a large number of genotypes, however, the squared correlation based on genotypic allele counts is equivalent to the *r*^2^ calculated from haplotype frequencies [36, 37]. Another limitation is that the phasing approaches for heterozygous polyploids usually need access to a reference genome, which is not available for sugarcane [3, 12].

Unlike plant species with simpler genomes like barley and wheat, segregation occurs in sugarcane within the first generation of a progeny generated through biparental crosses. As a result, genetic mapping has been restricted to single-dose markers. In this approach, a copy (dose) of a particular marker is present in either one or both parents, resulting in a 1:1 or 3:1 (presence: absence) ratio in the mapping population [5, 8, 9, 38, 39]. The challenge was assessing LD estimates in sugarcane since lower dosage markers only reflect a locus’s partial genetic information; nevertheless, the LD values assessed in this work are just a proxy for actual calculations.

The decay of LD over genetic distance was investigated by plotting pair-wise intra-chromosomal *r*^2^ values against the genetic distance (cM) between markers. The R package ggplot2 was used to visualise the results, including a locally estimated scatterplot smoothing (LOESS) line [40]. After analysing the distribution of the observed *r*^2^ values, the critical *r*^2^ value for all crop species in this study was set to 0.1, which refers to the minimum threshold for a significant association between two loci. The LD decay over genetic distance was determined as the mean distance associated with an empirical threshold of *r*^2^ = 0.1. The analysis was run with a range of different *r*^2^ thresholds.

### Algorithm

There are two main steps in the algorithm (Fig. 4) used in this study: i) Assigning an unmapped SNP to a chromosome; ii) estimating the SNP’s location within the assigned chromosome. For each unassigned SNP with MAF > 0.01, *r*^2^ between the unmapped and all mapped SNPs was estimated.

#### Testing algorithm performance

For each unassigned SNP, the previously mapped SNPs with the first and second highest *r*^2^ (> 0.1) estimates were identified, and if both mapped SNPs were on the same chromosome, the unassigned SNP was allocated to the same linkage group (or chromosome). To test the algorithm accuracy, groups of SNPs with an actual map position in each species had their position masked and were considered unassigned. Then, each “unassigned” SNP that could be allocated to a chromosome was positioned within a chromosome using a weighted average of mapped markers’ position, where the weight was determined by the LD estimates of the unpositioned SNP with mapped markers. The algorithm’s performance was assessed in terms of “Efficiency,” defined as the percentage of “masked” SNPs assigned to a chromosome, “Accuracy” as the percentage of SNPs allocated to the correct chromosome, and “Precision” as the difference in the distance between the known and assigned positions.

The algorithm was evaluated on various sizes of test sets of masked SNPs in sugarcane (~ 200), wheat (~ 900), and barley (~ 130) using random 20-fold cross-validation in which 20 random non-overlapping sets of masked SNPs were selected. All mapped SNPs were divided into 20 groups at random, with each unique group of SNPs masked (“unmapped”) and the remaining groups of SNPs classified as “mapped”.

## Data Availability

The R-code and datasets used (for sugarcane) and updated map using this methodology are made available on GitHub at this link https://github.com/SimmiSudhir/Linkage-Disequilibrium-Algorithm.git. The cultivar Q208 sugarcane genetic map is available on request from Karen.Aitken@csiro.au. The barley data can be accessed by contacting l.hickey@uq.edu.au and wheat data from Kai.Voss-Fels@hs-gm.de.
